# How Michigan Defines the Practice of Medicine

**Published:** 1899-11

**Authors:** 


### HOW MICHIGAN DEFINES THE PRACTICE OF MEDICINE.

THERE is a doubt in the minds of many people as to what constitutes the practice of medicine in its technical and legal sense. Some are of the opinion that if a person does not administer drugs in the attempt to cure disease he or she does not technically "practise," hence all faith healers, rubbers, masseurs, *et id genus doctores*, are exempt from the restriction that the law places around educated men and women who possess the doctorate degree and the state license to practise, both regularly obtained.

In the State of Michigan this question has lately been raised and the secretary of the state board of health referred it to the Attorney-General for an opinion. We take pleasure in publishing the opinion in full, hoping it may contribute to the solution of this vexed question.

### Every Person who Acts as a Physician Must Report Every Dangerous Contagious Disease to the Health Officer.

### OPINION OF THE ATTORNEY GENERAL.

Henry B. Baker, *Secretary State Board of Health, Capitol.*

*My Dear Sir*—Your letter of recent date received. You ask whether or not a person practising "a system known as christian science," is or is not a physician, or acting as a physician, and therefore liable, under section 1676 of Howell's statutes, as amended by act 11, laws of 1883, and act 150, public acts of 1895, for not reporting diseases to the health officer of the township, city or village, as required by law. Said act as amended requires that:

"Whenever any *physician* shall know that any person whom he is called to visit, or who is brought to him for examination, is infected with small-pox, cholera, scarlet fever, or any other disease dangerous to the public health, he shall immediately give notice thereof to the health officer of the township, city or village in which the sick person may be; and to the householder, hotel keeper, keeper of a boarding house, or a tenant within whose house or rooms the sick person may be. The notice to the officer of the board of health shall state the name of the disease, the name, age, and sex of the person sick, also the name of the physician giving the notice; and shall, by street and number, or otherwise, sufficiently designate the house or room in which said sick person may be. And every *physician and person acting as a physician*, who shall refuse or neglect immediately to give such notice, shall forfeit for each such offense a sum not less than ten nor more than fifty dollars," etc.

It is plain from the wording of the above section of the act that it applies to physicians as such, or persons acting as physicians. The question, therefore, hinges on whether or not a person employing "a system known as christian science" is or is not included under the above terms. If such a person is included under the term "physician" or "acting as a physician," he would be required to make the report, otherwise he would not.

I will first take up the construction of the word "physician." In section 3 of act 237, public acts of 1899, entitled:

"An act to provide for the examination, regulation, licensing, and registration of physicians and surgeons, and for the punishment of offenders against this law, and to repeal acts and parts of acts in conflict therewith."

I find it clearly defined who shall be physicians in this state, *i. e.*, who shall be allowed to practise medicine and surgery therein, requiring that all such persons shall pass a certain examination given by the board, together with other conditions, or be a graduate of certain medical schools, whereupon they shall be entitled to be registered, and receive a certificate of registration, which shall entitle them to practise medicine in Michigan.

Bouvier says:

"A physician is one *lawfully* engaged in the practice of medicine."

This is also the common and accepted meaning of the term. It follows, therefore, that unless a person has received such a certificate entitling him to lawfully practise, he is not a physician in the State of Michigan, and will be liable for holding himself out as such under section 7 of the act in question, which section reads, in part, as follows :

" Any person who shall practise medicine or surgery in this state, without first complying with the provisions of this act, shall be deemed guilty of a misdemeanor, and upon conviction thereof shall be punished by a fine of not more than one hundred dollars, or by imprisonment in the county jail for a period of not more than ninety days, or by both such fine and imprisonment, for such offense," etc.

Section 9 of act 237, also provides :

" When any person shall append the letters M. B. or M. D., or prefix the title " Dr." or doctor, or any other sign or appellation in a medical sense to his name, it shall be prima facie evidence of practising medicine and surgery within the meaning of this act."

Taking the definition of Bouvier, and the Encyclopedia of Law, that : "A physician is one lawfully engaged in the practice of medicine," together with sections 3,7 and 9 of act 237, it seems that the clear intention of the legislature is, that only such persons as have received the necessary certificate from the board of registration are to be considered physicians in the State of Michigan. It is my opinion, therefore, that a person employing "a system known as christian science," unless he be lawfully registered as a physician, is not a physician, and cannot be included under the term "physician," as used in the act under consideration.

This brings us to the words "acting as a physician," the question being, whether they or not would include a person practising "a system known as christian science." The evident intention of the legislature in adding this clause was to broaden the meaning of the section in order that it may include persons not physicians, and thus bring within its construction all those cases which might otherwise fail to be reported. The question is, then, who did the legislature intend to include in the words "acting as a physician?" I think that they are capable of a very wide meaning, and if it was the intention of the legislature to give them this wide meaning they should be so construed. The evil sought to be overcome by the statute is the danger to public health and life due to the failure of physicians and persons acting as physicians, to make a report of diseases which they undertake to treat, in order that the public may be warned, and such disease may be properly confined. If this was the intention of the legislature, which I think it was, it follows that any person who takes charge of and attempts to treat any of the contagious diseases mentioned in the statute, whatever the means, would be liable for not making the report as required by the act. This would include a person employing "a system known as christian science."

Yours respectfully,

(Signed) HORACE M. OREN, Attorney-General.

